# Supplementary material for: Multireference Equation-of-Motion Driven Similarity Renormalization Group: Theoretical Foundations and Applications to Ionized States
Source: J Chem Theory Comput. 2025 Aug 15;21(16):7903–19. doi: 10.1021/acs.jctc.5c00992 (PMC12392458; doi:10.1021/acs.jctc.5c00992)
Supplement: Supplementary file 2 [file ct5c00992_si_002.pdf]

Supporting information: Multireference equation-of-motion driven similarity renormalization group: theoretical foundations and applications to ionized states

Zijun Zhao,[1, a)] Shuhang Li,[1, b)] and Francesco A. Evangelista[1, c)]

Department of Chemistry and Cherry Emerson Center for Scientific Computation, Emory University, Atlanta, Georgia 30322, USA

a)Electronic mail: zijun.zhao@emory.edu

b)Electronic mail: shuhang.li@emory.edu

c)Electronic mail: francesco.evangelista@emory.edu

I. CHOICE OF ACTIVE SPACE AND REFERENCE ENERGIES

Here we document the active space choices for the molecules studied in Section IV.A. The CASSCF reference energies are also reported. All CASSCF calculations are converged to an energy threshold of 10^{-12} a.u. and a gradient norm of 10^{-10} a.u. The active space is specified by the number of core and active orbitals per irrep, ordered in the 'Cotton ordering', *i.e.*, [a_g, b_{1g}, b_{2g}, b_{3g}, a_u, b_{1u}, b_{2u}, b_{3u}] for the D_{2h} point group, and [a_1, a_2, b_1, b_2] for the C_{2v} point group. The atomic valence active space (AVAS) method[1] is used to select the initial active space orbitals as needed, and the AVAS subspace is specified for those cases.

TABLE S1. Choice of active spaces and references used in the EOM-DSRG computations. Reference CASSCF energies (E_{CAS}) in hartree (E_{h}).

Molecule	Core	Active	AVAS	E_{CAS} / E_{h}
C_2	[1,0,0,0,0,1,0,0]	[2,0,2,2,0,2,2,2]		-75.652069959610
C_2H_4 (eqm)	[1,0,0,0,0,1,0,0]	[3,0,1,2,0,3,2,1]	["C(2s)","C(2p)","H(1s)"]	-78.994626688870
C_2H_4 (str)				-77.945136269290
CO	[2,0,0,0]	[4,0,2,2]		-112.885952774734
CS	[4,0,1,1]	[4,0,2,2]		-435.438613682326
F_2 (eqm)	[1,0,0,0,0,1,0,0]	[2,0,1,1,0,2,1,1]		-198.777678643099
F_2 (str)				-198.753977458044
H_2CO (eqm)	[2,0,0,0]	[5,0,2,3]	["C(2s)", "C(2p)", "O(2s)", "O(2p)", "H(1s)"]	-114.014228155162
H_2CO (str)				-113.767278864429
H_2O (eqm)	[1,0,0,0]	[4,0,2,3]		-76.176803247525
H_2O (str)				-75.884613320071
HF (eqm)	[1,0,0,0]	[5,0,2,2]		-100.173027089660
HF (str)				-100.006723519719
N_2 (eqm)	[1,0,0,0,0,1,0,0]	[2,0,1,1,0,2,1,1]		-109.108676505452
N_2 (str)				-108.785572038754

For the spectroscopic constants of radicals, the active space is specified as follows:

- CN^-: core: [2,0,0,0], active: [4,0,2,2], AVAS subspace: ["C(2s)", "C(2p)", "N(2s)", "N(2p)"]

- CO: same as above
- N_2: same as above
- OH^-: core: [1,0,0,0], active: [3,0,2,2]

For the CN binding curve, the same active space as CN^- above is used.

All state-averaged calculations are performed using the same active space as above, using an ensemble of only the targeted states, with equal weights. Additional states of zero weight may be added to remedy the occasional root-flipping problem.

II. VERTIAL IONIZATION ENERGIES OF MOLECULES AT EQUILIBRIUM GEOMETRIES

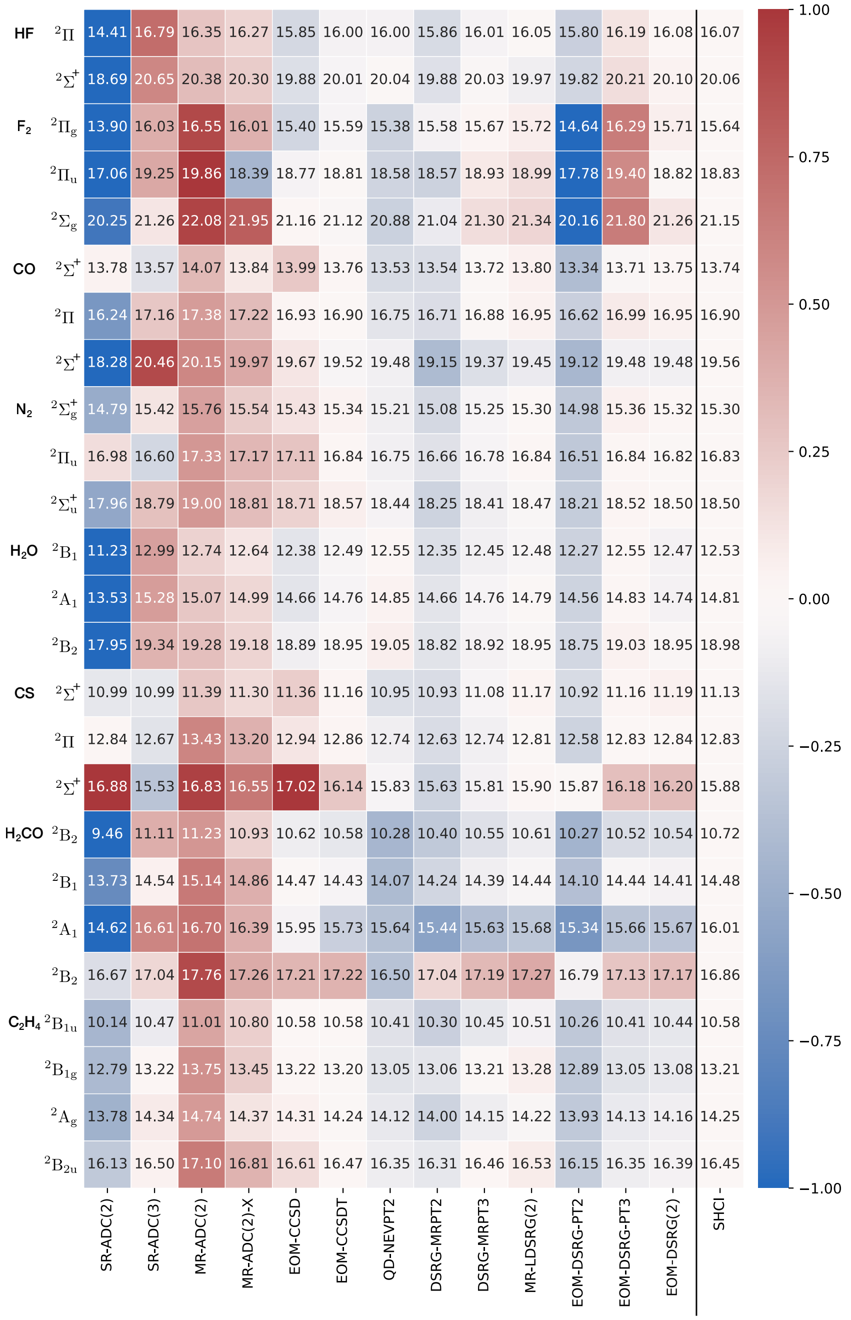

FIG. S1. Vertical ionization energies (IPs) of molecules at equilibrium geometries. The colors indicate the signed error (eV) of the IPs with respect to the SHCI values.

III. VERTIAL IONIZATION ENERGIES OF MOLECULES AT STRETCHED GEOMETRIES

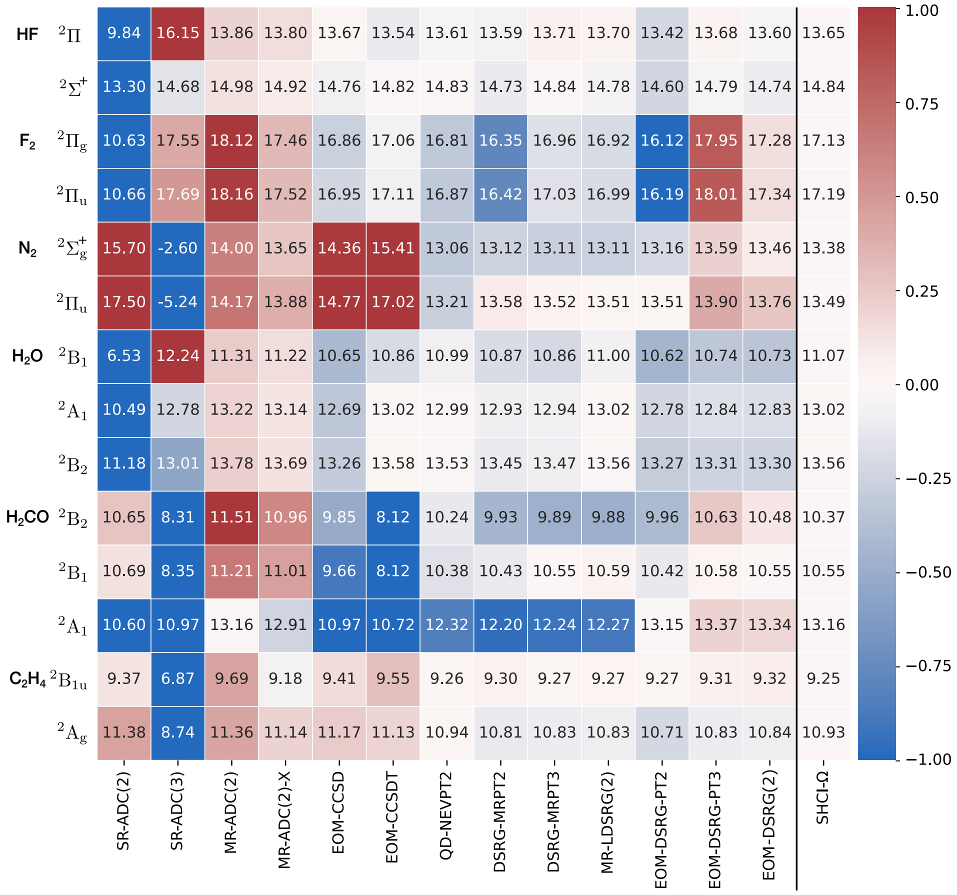

FIG. S2. Vertical ionization energies (IPs) of molecules at stretched geometries. The colors indicate the signed error (eV) of the IPs with respect to the SHCI values.

IV. OVERALL FLOW PARAMETER DEPENDENCE OF THE EOM-DSRG IPS

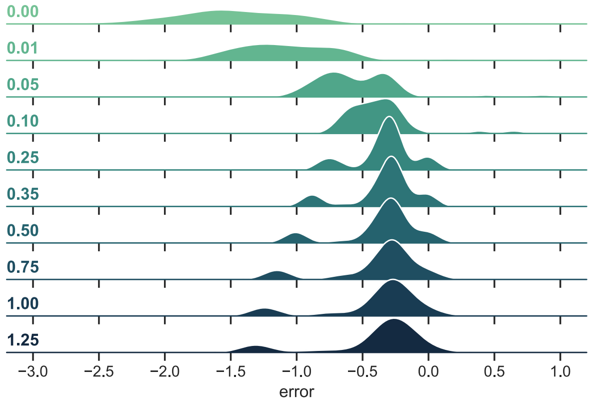

FIG. S3. Overall flow parameter dependence of the EOM-DSRG-PT2 IPs at both equilibrium and stretched geometries.

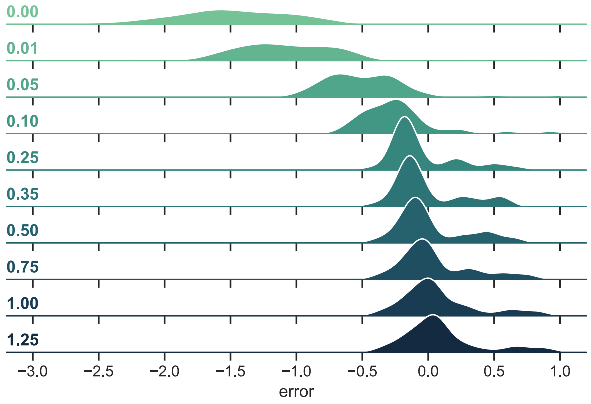

FIG. S4. Overall flow parameter dependence of the EOM-DSRG-PT3 IPs at both equilibrium and stretched geometries.

V. FLOW PARAMETER DEPENDENCE OF THE EOM-DSRG IPS AT EQUILIBRIUM GEOMETRIES

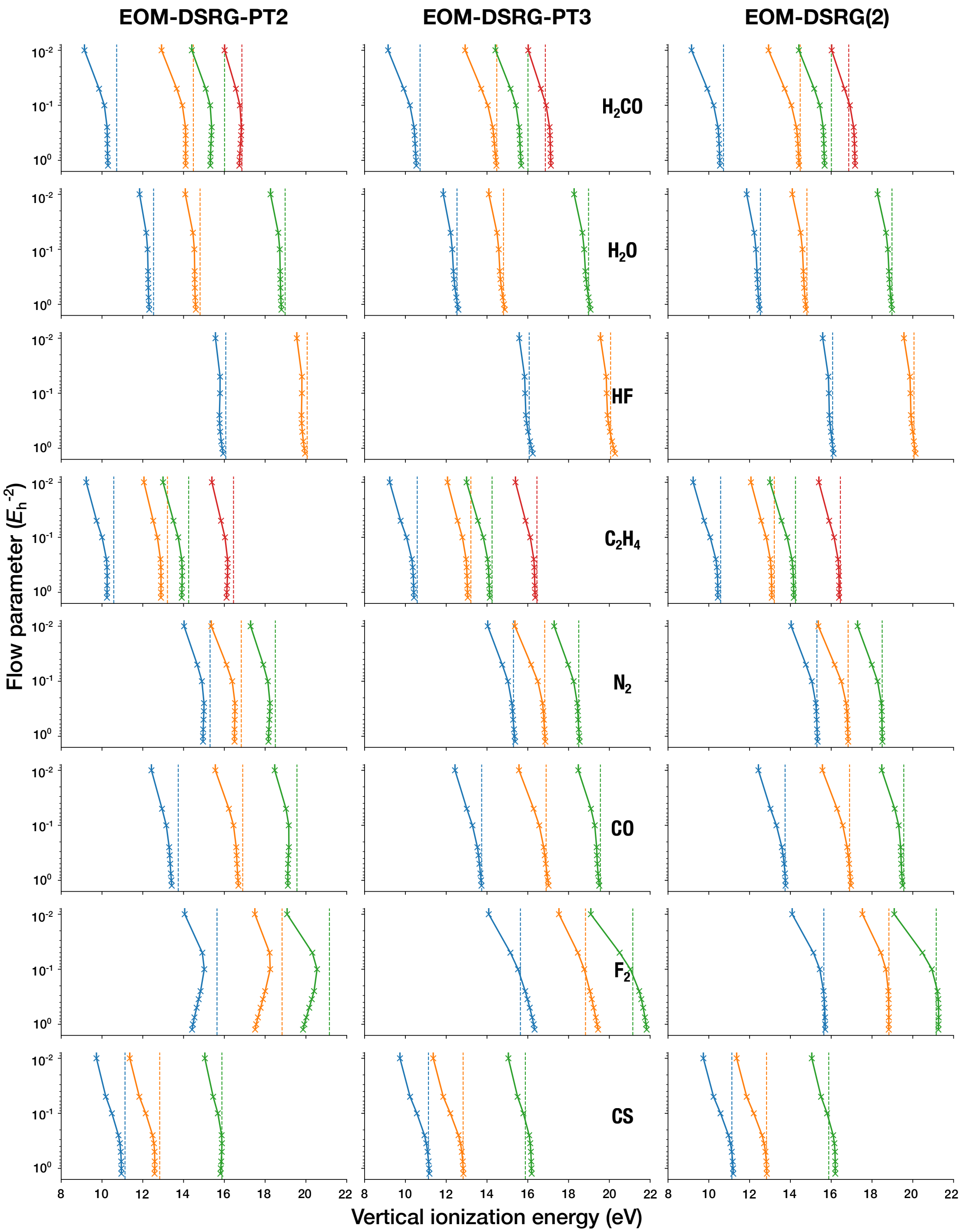

FIG. S5. Dependence of the ionization potentials (IPs) on the flow parameter.

VI. FLOW PARAMETER DEPENDENCE OF THE EOM-DSRG IPS AT STRETCHED GEOMETRIES

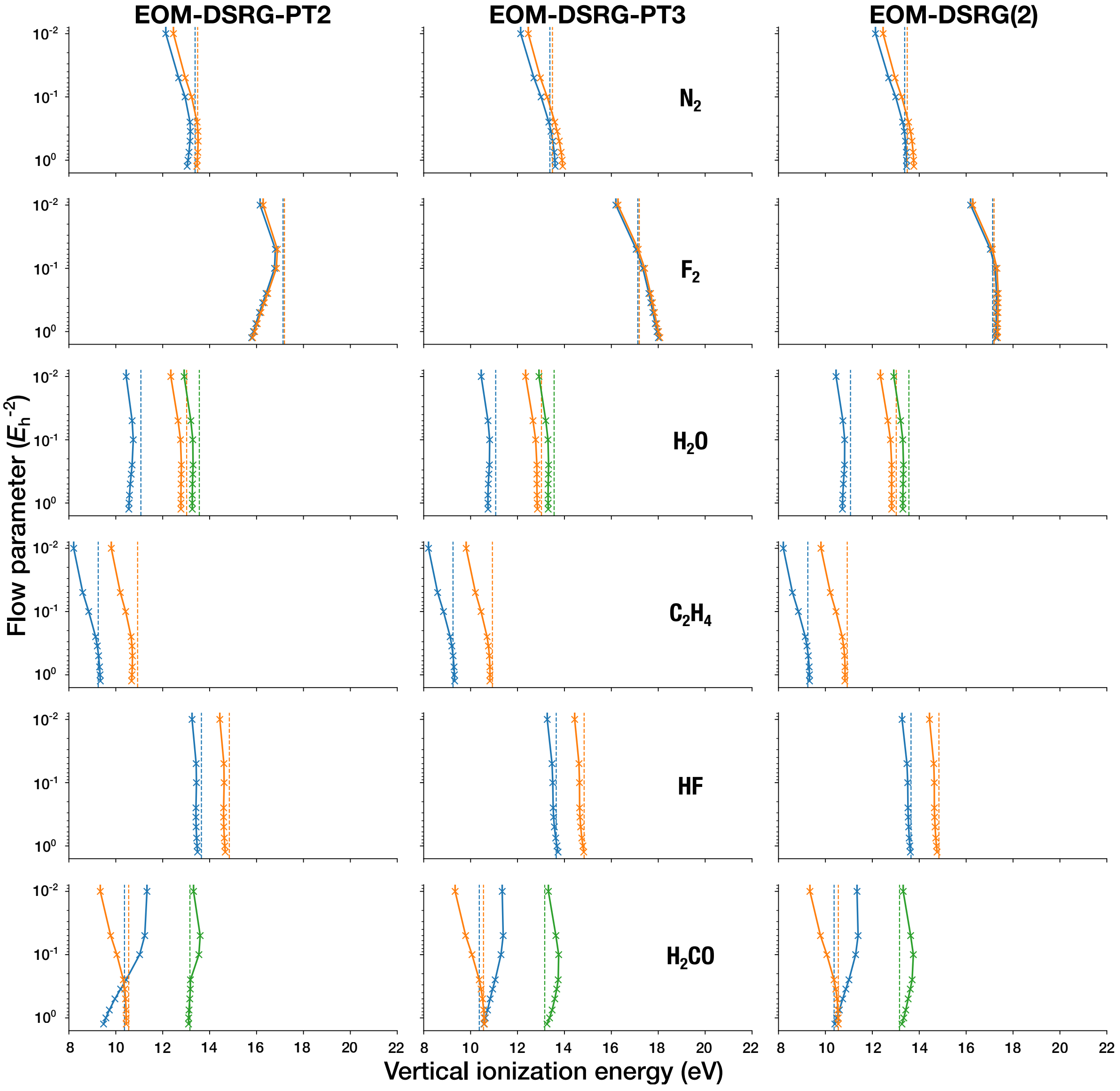

FIG. S6. Dependence of the ionization potentials (IPs) on the flow parameter.

VII. SPECTROSCOPIC CONSTANTS OF SELECT RADICALS

A. Equilibrium bond lengths

TABLE S2. Comparison of of equilibrium bond lengths (r_e, Å) for select low-lying electronic states of various radicals. All theoretical values other than those from EOM-DSRG are from Saeh and Stanton,[2] experimental values are from Huber and Herzberg.[3]

Molecule	State	UHF-CCSD	UHF-CCSD(T)	EOMIP-CCSD	EOMIP-CCSD*	EOM-DSRG	Expt
OH	$X\ ^2\Pi$	0.9673	0.9694	0.9647	0.9688	0.9698	0.9697
OH	$A\ ^2\Sigma^+$	1.0047	1.0061	0.9966	1.0045	1.0063	1.0121
CN	$X\ ^2\Sigma^+$	1.1640	1.1707	1.1650	1.1710	1.1759	1.1718
CN	$A\ ^2\Pi$	1.2284	1.2350	1.2221	1.2294	1.2383	1.2333
CN	$B\ ^2\Sigma^+$	1.1368	1.1499	1.1415	1.1464	1.1547	1.150
N_2^+	$X\ ^2\Sigma_g^+$	1.1110	1.1192	1.1073	1.1132	1.1208	1.1164
N_2^+	$A\ ^2\Pi_u$	1.1674	1.1765	1.1570	1.1743	1.1806	1.1750
N_2^+	$B\ ^2\Sigma_u^+$	1.0623	1.0744	1.0647	1.0687	1.0778	1.0742
CO^+	$X\ ^2\Sigma^+$	1.1104	1.1162	1.1086	1.1170	1.1193	1.1151
CO^+	$A\ ^2\Pi$	1.2395	1.2471	1.2362	1.2336	1.2506	1.2437

B. Harmonic vibrational frequencies

TABLE S3. Comparison of harmonic vibrational frequencies (ω_e, cm^{-1}) for select low-lying electronic states of various radicals. All theoretical values other than those from EOM-DSRG are from Saeh and Stanton,[2] experimental values are from Huber and Herzberg.[3]

Molecule	State	UHF-CCSD	UHF-CCSD(T)	EOMIP-CCSD	EOMIP-CCSD*	EOM-DSRG	Expt
OH	$X\ ^2\Pi$	3796	3763	3861	3763	3752	3738
OH	$A\ ^2\Sigma^+$	3295	3282	3425	3300	3257	3179
CN	$X\ ^2\Sigma^+$	2178	2134	2165	2115	2070	2069
CN	$A\ ^2\Pi$	1877	1841	1927	1874	1809	1813
CN	$B\ ^2\Sigma^+$	2375	2212	2339	2276	2188	2164
N_2^+	$X\ ^2\Sigma_g^+$	2292	2215	2352	2277	2200	2207
N_2^+	$A\ ^2\Pi_u$	2006	1922	2095	1924	1891	1904
N_2^+	$B\ ^2\Sigma_u^+$	2633	2486	2633	2582	2444	2420
CO^+	$X\ ^2\Sigma^+$	2313	2293	2315	2242	2220	2214
CO^+	$A\ ^2\Pi$	1626	1573	1651	1672	1551	1562

C. Adiabatic electronic excitation energies

TABLE S4. Comparison of adiabatic electronic excitation energies (T_e, cm^{-1}) for select low-lying electronic states of various radicals. All theoretical values other than those from EOM-DSRG are from Saeh and Stanton,[2] experimental values are from Huber and Herzberg.[3]

Molecule	Transition	UHF-CCSD	UHF-CCSD(T)	EOMIP-CCSD	EOMIP-CCSD*	EOM-DSRG	Expt
OH	$X\ ^2\Pi \rightarrow A\ ^2\Sigma^+$	33561	33472	33709	33499	32602	32684
CN	$X\ ^2\Sigma^+ \rightarrow A\ ^2\Pi$	7704	8695	9708	8831	9129	9245
CN	$X\ ^2\Sigma^+ \rightarrow B\ ^2\Sigma^+$	26840	26865	28115	26262	25570	25752
N_2^+	$X\ ^2\Sigma_g^+ \rightarrow A\ ^2\Pi_u$	7198	8969	11228	8144	8782	9167
N_2^+	$X\ ^2\Sigma_g^+ \rightarrow B\ ^2\Sigma_u^+$	27082	26322	25490	26025	25383	25461
CO^+	$X\ ^2\Sigma^+ \rightarrow A\ ^2\Pi$	19281	20034	19454	21367	20529	20733

VIII. CN BINDING CURVE FOR PT2 AND PT3

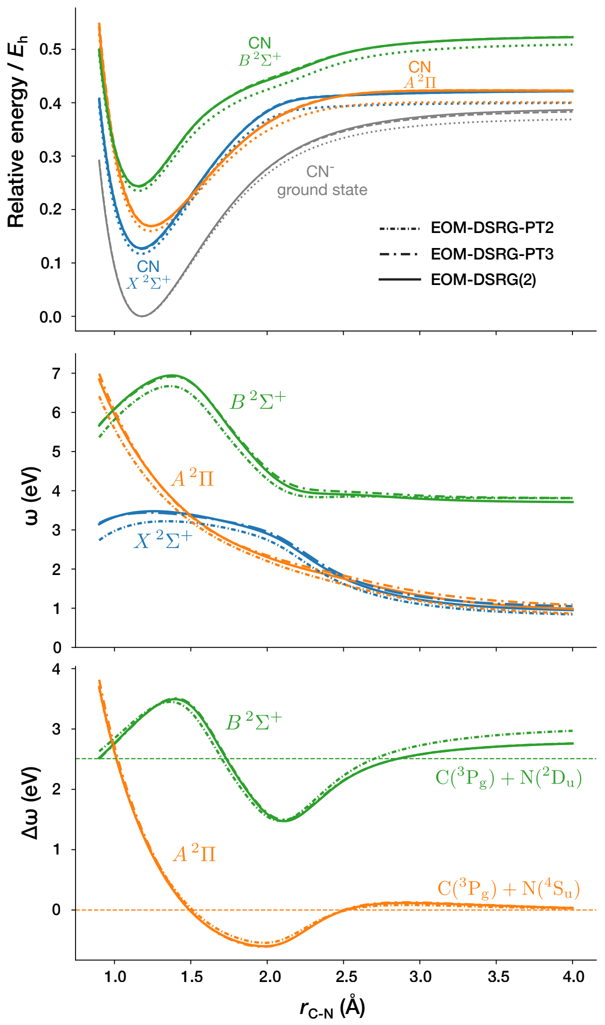

FIG. S7. Similar to Fig. 10 in the main text, but comparing IP-EOM-DSRG-PT2/3 to IP-EOM-DSRG(2).

REFERENCES

[1]E. R. Sayfutyarova, Q. Sun, G. K.-L. Chan, and G. Knizia, J. Chem. Theory Comput. **13**, 4063 (2017).

[2]J. C. Saeh and J. F. Stanton, J. Chem. Phys. **111**, 8275 (1999).

[3]K. P. Huber and G. Herzberg, *Molecular Spectra and Molecular Structure* (Springer US, Boston, MA, 1979).
